# Improving medication error classification using a reasoning large language model

**DOI:** 10.1093/jamiaopen/ooag004

**Published:** 2026-01-24

**Authors:** Anders Krifors, Theodor Beskow, Magnus Jonsson, Karl-Johan Lindner, Jenny Calås, Veronica Arwsbo, Kristian Sandström, Christer Norström

**Affiliations:** Centre for Clinical Research Västmanland, Uppsala University, Västerås Hospital, 721 89 Västerås, Sweden; Unit of Infectious Diseases, Department of Medicine Huddinge, Karolinska Institutet, 171 77 Stockholm, Sweden; Norrvy Intelligence AB, 117 59 Stockholm, Sweden; Norrvy Intelligence AB, 117 59 Stockholm, Sweden; Department of Pharmacy, Region Västmanland, 721 89 Västerås, Västmanland County, Sweden; Department of Pharmacy, Region Västmanland, 721 89 Västerås, Västmanland County, Sweden; Department of Pharmacy, Region Västmanland, 721 89 Västerås, Västmanland County, Sweden; School of Innovation, Design and Engineering, Mälardalen University, 721 23 Västerås, Sweden; School of Innovation, Design and Engineering, Mälardalen University, 721 23 Västerås, Sweden

**Keywords:** medication errors, natural language processing, artificial intelligence, incident reporting, patient safety

## Abstract

**Objectives:**

To assess the performance of a reasoning large language model (LLM) in identifying medication errors in medical incident reports.

**Materials and Methods:**

OpenAI’s O4-mini LLM was adapted using prompt engineering on 75 000 anonymized incident reports from the Västmanland region of Sweden (2019-2024). To guide the prompt design, we used a subset of 2434 reports, which were manually reclassified by pharmacists as medication-related or not. For validation, 200 reports (January 2024-March 2024) were independently classified by 2 pharmacists to establish a reference classification. Moreover, the LLM performed binary classification, with concordance rates measured against the expert consensus.

**Results:**

The LLM achieved a concordance rate of 96.0% (192/200; 95% CI, 92.3-98.3) with expert classification. Eight cases (4.0%) showed disagreements, primarily due to linguistic ambiguity or context-dependent interpretation. Five cases involved pharmacists classifying reports as non-medication-related, while the LLM classified them as medication-related, with the reverse in 3 cases. Subcategorization accuracy was 76.5%.

**Discussion:**

The LLM showed expert-level performance, outperforming existing automated methods. Thus, its integration into incident reporting systems might improve the efficiency, accuracy, and consistency of patient safety monitoring.

**Conclusion:**

This validated AI-driven method can be integrated directly into clinical informatics workflows, enabling healthcare organizations to rapidly and consistently identify medication errors, ultimately enhancing patient safety outcomes.

## Introduction

Medication errors represent a significant patient safety issue, accounting for approximately 10% of all cases of preventable patient harm.^1^ Observational studies have reported error rates ranging between 11.7% and 13.2% of all in-hospital medication administrations.^2^^,^^3^ However, incident reporting systems only capture around 1% of these errors.^4^ This discrepancy largely results from systematic under-reporting and inconsistent classification practices. Consequently, relying solely on incident reporting data substantially underestimates the true scale of medication errors, undermining the opportunities for targeted preventive strategies.

Medication error classification relies heavily on subjective assessments by healthcare professionals whose expertise and experience vary considerably, leading to inconsistent and unreliable categorizations. The accurate classification of medication errors is a complex process that requires substantial domain expertise and contextual understanding. Many incident reporting systems lack standardized protocols and terminology, such as the taxonomy from the National Coordinating Council for Medication Error Reporting and Prevention,^5^ to systematically classify medication errors. Lindner et al. have reported significant improvements in error classification quality through structured reclassification by clinical pharmacists.^6^ However, this approach is resource-intensive, limiting its implementation at larger scales. Previous attempts to standardize medication error classification have been associated with several challenges arising from high variability and inconsistencies across clinical settings.^7^ A recent systematic review further emphasized the need for more robust, standardized classification frameworks.^8^ Thus, efficient, reliable, and scalable classification methods are urgently warranted.

Machine learning has been used to address these demands. Fong et al^9^ employed a model to categorize medication errors in the FDA’s adverse event database, achieving a 92% accuracy; however, the system struggled when dealing with unclear or varied error descriptions. Moreover, Härkänen et al. used an AI-driven system to analyze incident reports at a Finnish university hospital, successfully identifying critical risk areas, including verification, documentation, and communication. However, the findings were not validated against expert classifications. Although these AI-based methods can improve classification accuracy compared to non-expert assessments, their practical effectiveness relies heavily on the availability of high-quality data and formatting. Large language models (LLMs), with advanced capabilities in interpreting and processing unstructured text, have emerged as a promising technology for addressing the challenges of previously used methods. LLMs have enhanced the understanding of language context, enabling a more consistent, accurate, and efficient error classification. Although previous AI-based classification methods exist, they lack rigorous validation against expert pharmacist classifications, limiting their clinical applicability. This study aimed to assess the performance of a reasoning LLM in classifying medication error reports.

The study was conducted in the Västmanland region of Sweden, encompassing healthcare facilities with approximately 550 beds and administering around one million injections or infusions and 1.7 million oral doses annually. Each year, about 13 000 incidents are recorded in the Synergi Life incident reporting system (DNV, Bergen, Norway), with medication errors accounting for 5%-6% of all incidents. The system captures various incident types beyond medication errors but lacks standardized, systematic error classification procedures. Typically, medication errors are classified locally at the clinical level without hospital-wide oversight, with reporting open to all healthcare staff.

## Methods

This study used OpenAI’s reasoning model O4-mini (OpenAI, San Francisco, CA), which was hosted securely via Microsoft Azure (Microsoft Corporation, Redmond, WA). The model is a compact and efficient variant of OpenAI’s GPT-4o architecture, which is optimized for high-speed inference and low resource consumption while retaining strong reasoning capabilities.

The model underwent fine-tuning using prompt engineering techniques to enhance its accuracy and contextual understanding specific to medication errors. The main training dataset comprised 75 000 fully anonymized incident reports, which were classified by over 500 contributors, introducing substantial variability in the data. Of these, 2434 reports had been systematically reclassified by expert pharmacists into non-medication errors or 8 predefined categories: prescription, dispensing, storage/inventory, preparation, administration, transcription, monitoring, and other. To ensure consistency and reliability, pharmacists reached consensus on categorization through collaborative discussions that resolved initial disagreements. The dataset was used for LLM prompt design and represented various incident types spanning multiple hospital departments and healthcare contexts. For external validation, we selected 200 additional medication error reports submitted between January and March 2024, ensuring no overlap with the training dataset. Two expert hospital pharmacists with specialized training and extensive experience in medication safety analysis independently classified these reports, establishing a reference to validate the performance of the LLM. The primary outcome was the concordance between the LLM classifications and expert reference. The concordance rates were measured as the proportion of cases where LLM classifications matched the expert consensus. Exact binomial methods were used to measure 95% CIs for the observed concordance rate. The secondary analysis involved sub-categorizing reports into prescription, dispensing, storage/inventory, preparation, administration, transcription, monitoring, and other.

Ethical approval was obtained from the Swedish Ethical Review Authority (Dnr 2025-00121-0). In accordance with the General Data Protection Regulation laws, all incident reports used in the present study were fully anonymized prior to analysis, ensuring no identifiable patient or staff information was included. The dataset, comprising 75 000 incident reports and a validation subset of 200 reports, was handled securely within a Microsoft Azure environment.

## Results

Of the 200 validation cases, the LLM accurately classified 192 reports, reaching an overall concordance rate of 96.0% (192/200) with the expert reference standard. Using the exact binomial methods, the 95% CI for the observed concordance rate of 96.0% was calculated to be 92.3% to 98.3%. All classifications included detailed reasoning provided by the model.

Eight cases (4.0%) showed a disagreement between the LLM and expert classifications. The post hoc analysis of these discordant cases revealed that most disagreements stemmed from linguistic ambiguity or context-dependent interpretation. In 5 cases, pharmacists classified the reports as non-medication-related, whereas the LLM did the opposite; an opposite scenario was observed in the remaining 3 cases. Two cases involved misinterpreted terms, such as excluding chlorhexidine as a medication or misreading the name of a sales catalogue as a drug. In 2 cases, the LLM strictly applied the prompt rules, overlooking broader organizational or communication aspects that pharmacists deemed relevant, and the reports were classified as medication errors. Moreover, one case involved a technical error with a downstream medication risk, leading to different yet reasonable conclusions. The remaining discrepancies were ambiguous and not easily classifiable, suggesting that an improved prompt design and hybrid human-AI approaches could further enhance classification accuracy, especially for edge cases.

The model achieved 76.5% concordance with expert subclassification (153/200 cases).

## Prompt design

We applied and compared several prompt strategies in a systematic development process:

- Prompt Strategy 1: Learning From Examples
  The model was initially exposed to examples of correct and incorrect classifications based on existing manual annotations. However, the model’s performance was hindered by the inconsistency of the training data, achieving a concordance rate of approximately 50% with manual classifications.
- Prompt Strategy 2: Rule-Based Prompting
  An explicit prompt was developed based on the classification rules derived from data patterns and institutional guidelines. It was a promising approach; however, the model’s performance remained insufficient, with a concordance rate of approximately 75%. A simplified version relying solely on the model’s internal knowledge was also assessed but performed worse, with a concordance rate of 67%.
- Prompt Strategy 3: Encoding Tacit Knowledge
  A structured prompt was developed using informal guidelines, heuristics, and knowledge from experienced hospital pharmacists. This approach incorporated expert judgment patterns and contextual understanding that had not been formally documented. This approach yielded higher agreement with the test data, with a concordance rate of 75% and several disagreements attributable to model outputs that were arguably more guideline-consistent compared to human classifications.
- Prompt Strategies 4-5: Critic Model Integration
  A secondary “critic” prompt was designed to review each classification, providing justifications and flagging disagreements. When the critic disagreed with the initial classification, disputed cases were manually reviewed by expert pharmacists, and the original prompt was refined based on this feedback. This approach incorporated a built-in quality assurance mechanism for continual refinement.

The final prompt incorporated comprehensive classification rules, contextual guidelines, and expert reasoning. This prompt was structured in a format that allowed consistent decision-making. The complete prompt specification included detailed classification categories, decision trees, and procedures for handling edge cases. The prompt is in Swedish and can be provided upon request.

## Method for increased reliability

Recent research has shown that self-consistency approaches—wherein multiple reasoning paths are generated and aggregated—can significantly improve response quality (10). Moreover, iterative refinement methods, wherein the model re-evaluates and improves its outputs, have also shown promising results (11). Notably, reliability-enhancing techniques for LLMs are a rapidly evolving area of study.

In the present study, a combination of 2 fundamental techniques was used to increase response reliability: (1) multiple executions of the same prompt, which was referred to as the *classifier prompt*, and (2) a secondary prompt designed to critically assess the response after the execution of each classifier prompt. This approach provides both a repeated evaluation and an internal consistency check, which helps derive a measure of reliability.

**Figure 1.**
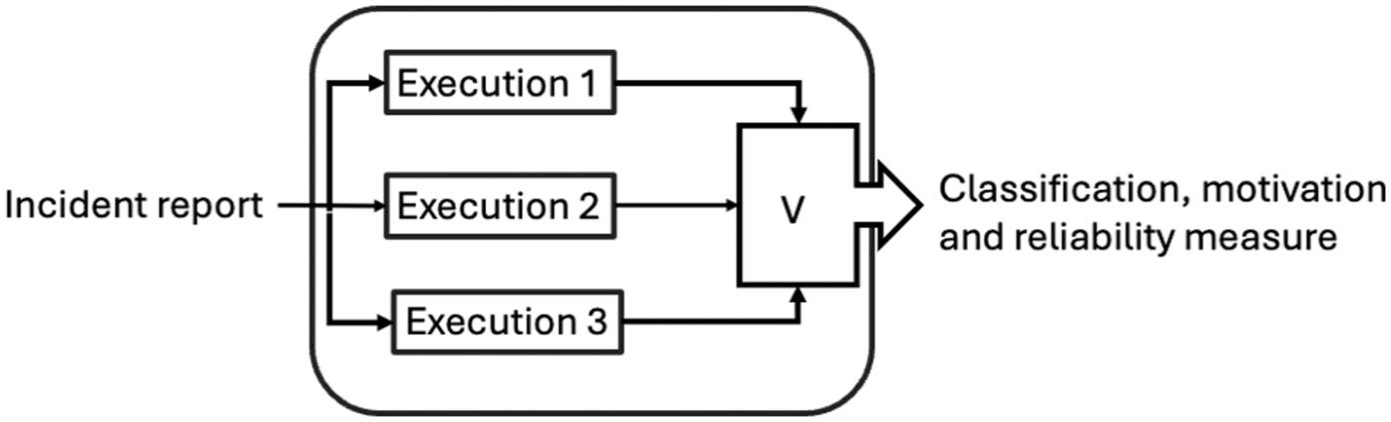
The architecture used for validation. Each execution includes 2 prompts: the classifier prompt and the critical assessment prompt.

The reliability measure was calculated by determining the proportion of prompts that yielded the same classification results. Figure 1 shows the architecture used for validation. Each execution comprised both the classifier prompt and its corresponding critical assessment, resulting in 6 classification outputs per incident.

If all 6 outputs provided the same classification, the reliability was 100%. In cases where the responses were evenly split, such as a 3-3 tie, the reliability was considered to be 50%. Such ties might indicate ambiguity in either the incident report or the specification used for classification.

When integrating the approach into a real system, the process design should define the conditions that require manual intervention. For example, a 4-2 or 3-3 split in the classification responses might indicate ambiguity in the input or the specification, warranting a manual review. On the other hand, if the outcome is 5-1 or 6-0, the response can be considered reliable and trusted without the need for further intervention.

## Discussion

This study reported that the O4-mini LLM achieved a concordance rate of 96.0% (95% CI, 92.3-98.3) with expert pharmacist classifications for identifying medication errors. The performance of our model surpassed the reported accuracy of previous automated methods (12–14), indicating its high potential in classifying medication error incidents. Regression or traditional machine learning approaches frequently require extensive manual preprocessing and struggle with language inconsistencies and irregular formatting, whereas the O4-mini LLM effectively interpreted complex medical terminology, abbreviations, and incomplete data. LLMs are at the forefront of AI development, with improved versions released frequently. This technology has had a significant impact across various sectors, and the present study demonstrated that LLMs also excel in classification tasks. Integrating O4-mini into incident reporting systems, such as Synergi Life, could facilitate real-time error classification, allowing pharmacists to prioritize root-cause analysis over manual sorting. Alternatively, a hybrid system, wherein O4-mini generates initial classifications for verification by a clinician, could further improve accuracy, streamline workflows, and build clinicians’ trust. Accelerated identification of systemic issues can reduce both patient harm and associated healthcare costs, which are estimated to reach approximately $30.1 billion annually in the US and €79 billion in Europe due to medication error harm (15).

The model development process integrated a critical-thinking model and justifications for each classification decision, allowing clinicians to understand the reason behind every categorization. It established a framework for prompting models for other classification tasks beyond medication errors. Our process formalized expert judgment patterns and contextual understanding that had not been previously documented, thereby effectively capturing years of pharmacist expertise.

For the subclassification task, the model agreed with experts in 76.5% of cases (153/200). This is a strong result given that subclassification requires distinguishing between closely related medication-process categories, which often involves complex and context-dependent judgment.

However, our study had several limitations. It was conducted in a single region of Sweden, which might affect the generalizability of the results due to variations in reporting practices and electronic health record systems across different regions. A recent review highlighted challenges in implementing LLMs across healthcare systems, particularly in terms of interoperability and practice variability, emphasizing the need for local validation (16). Moreover, although the validation sample of 200 cases provided robust initial data, larger and multi-site studies are needed to verify our results. Moreover, the closed-source design of O4-mini limits transparency concerning its training and decision-making processes, potentially affecting interpretability and clinician trust. This may also affect reproducibility and long-term sustainability. To address this, we designed our approach to rely only on prompt instructions rather than model fine-tuning, so the same process can be used with newer and more powerful LLMs as they become available. Furthermore, reliance on historical data might lead to an inadequate capture of the current medication practices, posing risks of misclassification in novel clinical contexts. A risk of model-generated hallucinations is also present, emphasizing the need for frequent validations. However, the classification motivation provided by the model in combination with a hybrid implementation might mitigate these risks.

The implementation of AI in clinical practice requires careful consideration of ethical principles and adherence to regulatory frameworks, such as the EU AI Act (17). Our approach aligned with the current recommendations for the responsible use of AI in clinical decision support, emphasizing the importance of transparency and expert validation throughout the development process (18). Furthermore, collaboration between clinicians and AI developers will be crucial in this fast-moving field.

Our findings indicated that LLMs, guided by expertly designed prompts, can automate the classification of medication-related incident reports with model performance matching or surpassing that of human experts. To the best of our knowledge, our approach achieved the highest concordance in classifying medication errors to date, indicating a significant advance in patient safety methodology. Our method provided a scalable solution to improve consistency, reduce manual workload, and enhance the effectiveness of incident reporting systems.

Future research should focus on assessing the practical feasibility and impact of implementing our model in real-world healthcare settings. The key areas for exploration include clinician trust and acceptance, integration into existing clinical workflows, and measurable improvements in patient safety outcomes. Validation of our model across diverse healthcare contexts and evaluation of its applicability to other incident types are essential to validate its effectiveness.

In conclusion, our findings provided a theoretical basis for utilizing LLMs to enhance medication safety surveillance systems, with the potential to transform how healthcare organizations identify, classify, and address medication-related incidents at scale.

## Supplementary Material

ooag004_Supplementary_Data

## Data Availability

The data used in this study originate from anonymized incident reports collected within Region Västmanland. Due to healthcare data protection regulations, the data are not publicly available. Requests for access may be considered on a case-by-case basis and require appropriate ethical approvals.

## Code availability

The prompt specification is provided in Supplementary File 1 and may be used for academic and non-commercial purposes.
